# ntJoin: Fast and lightweight assembly-guided scaffolding using minimizer graphs

**DOI:** 10.1093/bioinformatics/btaa253

**Published:** 2020-04-20

**Authors:** Lauren Coombe, Vladimir Nikolić, Justin Chu, Inanc Birol, René L Warren

**Affiliations:** Canada’s Michael Smith Genome Sciences Centre, BC Cancer, Vancouver, BC V5Z 4S6, Canada

## Abstract

**Summary:**

The ability to generate high-quality genome sequences is cornerstone to modern biological research. Even with recent advancements in sequencing technologies, many genome assemblies are still not achieving reference-grade. Here, we introduce ntJoin, a tool that leverages structural synteny between a draft assembly and reference sequence(s) to contiguate and correct the former with respect to the latter. Instead of alignments, ntJoin uses a lightweight mapping approach based on a graph data structure generated from ordered minimizer sketches. The tool can be used in a variety of different applications, including improving a draft assembly with a reference-grade genome, a short-read assembly with a draft long-read assembly and a draft assembly with an assembly from a closely related species. When scaffolding a human short-read assembly using the reference human genome or a long-read assembly, ntJoin improves the NGA50 length 23- and 13-fold, respectively, in under 13 m, using <11 GB of RAM. Compared to existing reference-guided scaffolders, ntJoin generates highly contiguous assemblies faster and using less memory.

**Availability and implementation:**

ntJoin is written in C++ and Python and is freely available at https://github.com/bcgsc/ntjoin.

**Supplementary information:**

Supplementary data are available at *Bioinformatics* online.

## 1 Introduction

Producing highly contiguous assemblies enables important downstream research such as genetic association studies and *cis*-regulatory element analysis (Rice and Green, 2019). However, while the advancement of single molecule sequencing data such as linked reads and long reads has shown great promise in improving *de novo* genome assembly quality (Shafin *et al.*, 2019; Weisenfeld *et al.*, 2017), most draft assemblies are still not achieving chromosome-scale completeness.

For some draft genomes, more contiguous assemblies may be available for a different individual of the same species or even a closely related species. In this case, sequence synteny between the assemblies can be leveraged for assembly-guided scaffolding. For example, while long-read assemblies can generate contiguous draft genomes, the high error rates of the reads negatively impact the base quality, hindering gene annotation (Watson and Warr, 2019). Polishing using short reads is often used to improve the base-pair accuracy of the assemblies (Rice and Green, 2019; Warren *et al.*, 2019; Watson and Warr, 2019). An alternative approach to this polishing step is to assemble short reads separately and scaffold the short-read assembly using a long-read assembly, producing an assembly on par with the contiguity and structure of the long-read assembly and the base-pair accuracy of the short-read assembly.

Existing reference-guided scaffolders such as Ragout (Kolmogorov *et al.*, 2018) and Ragoo (Alonge *et al.*, 2019) rely on alignments of the draft assembly to a reference assembly; Ragout utilizes Progressive Cactus (Armstrong *et al.*, 2019) for large genomes, while Ragoo depends on minimap2 (Li, 2018) for the task. The use of minimizer sketches in tools such as minimap2 is very effective in compactly representing genome sequences. Instead of storing every word of size k (k-mer) from the input sequences, only a chosen set of k-mers or hash values (‘minimizers’) are retained, greatly reducing the computational cost of sequence data storage and manipulation (Roberts *et al.*, 2004).

Here, we introduce ntJoin, an assembly-guided scaffolder, which uses a lightweight, alignment-free mapping strategy in lieu of alignments to quickly contiguate a target assembly using one or more references.

## 2 Materials and methods

Given the input target and reference sequence(s) in fasta format, ntJoin first creates an ordered minimizer sketch for each of the supplied sets of sequences, as described previously (Roberts *et al.*, 2004) (Supplementary Figs S1 and S2). ntJoin then uses the ordered minimizer sketches from each input to build a single undirected graph that facilitates a lightweight mapping between them. In this graph, each node is a minimizer, and edges are created between minimizers that are adjacent in at least one of the ordered sketches. The edge weight is a measure that is used to place more emphasis on connections in certain input assemblies. Edge weights represent the sum of the user-specified weights of each input that supports that edge.

The graph is then subjected to a series of filtering steps. First, a global edge weight threshold is applied. Next, branching nodes (nodes with degree > 2) are identified, and incident edges are filtered with an increasing edge weight threshold until the degree of that node drops to <3. Generally, the weight of the reference is higher than the target assembly, causing these edges to be prioritized and results in fitting the target assembly to the reference structure. The filtering of incident edges of branch nodes results in the graph being a set of connected components, each of which is a linear path of minimizer nodes. The sequences of minimizers in the linear paths are then translated to ordered and oriented contig paths, which describe the final output scaffolds (Supplementary Figs S1–S3). This graph-based method allows the algorithm to perform misassembly correction in addition to scaffolding the input contigs based on the input reference assembly, as contigs can be broken at putative misassemblies in the default mode. If the user does not want the input contigs to be cut when fitting the reference sequence, the option ‘no_cut=True’ can be specified.

The final, scaffolded target assembly in fasta format is the main output of ntJoin. In addition, the details of how the target assembly was scaffolded including orientation, order and gap sizes are described in an output ‘path’ file, and, optionally, an agp file (option ‘agp=True’). Finally, the minimizer graph is output in ‘dot’ format, which gives all nodes and edges in the graph as well as the edge weights indicating the level of assembly support.

Each assembly was evaluated for contiguity and correctness using QUAST (v5.0.2;--scaffold-gap-max-size 100 000 --large) (Mikheenko *et al.*, 2018). This scaffold gap parameter setting results in gap size inconsistencies over 100 kb being classified as ‘extensive misassemblies’.

Detailed methods are available online.

## 3 Results and discussion

**Fig. 1.**
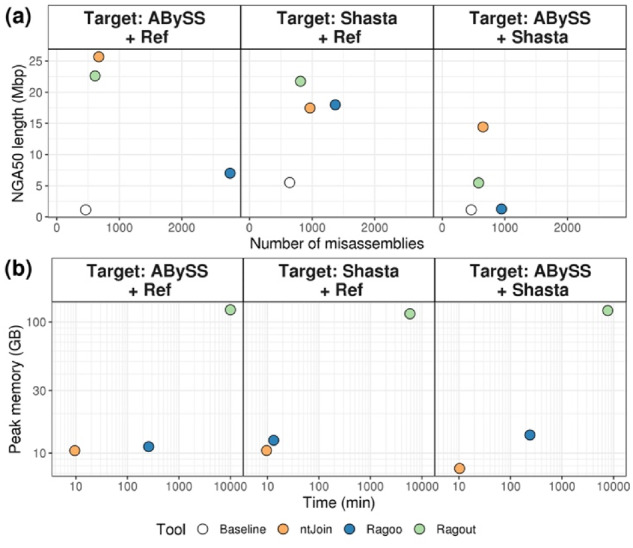
Comparing (**a**) the contiguity, correctness and (**b**) benchmarking results of ntJoin (orange), Ragoo (blue) and Ragout (green) runs on various *H.sapiens* (NA12878) assemblies on (a) linear and (b) log–log scale. The reference genomes are the human reference genome (‘Ref’) and an ntEdit-polished Shasta assembly (‘Shasta’). The target assemblies being improved are a NA12878 ABySS assembly scaffolded with MPET data (‘ABySS’), and an ntEdit-polished Shasta assembly (‘Shasta’). The ‘Baseline’ statistics are shown for the corresponding target assemblies prior to scaffolding in each panel of (a)

We first tested ntJoin using various draft and reference-grade *Caenorhabditis elegans* and *Homo sapiens* assemblies (Supplementary Tables S1 and S2). Compared to Ragout and Ragoo, ntJoin generally produces assemblies with a higher NGA50 length (length that captures at least 50% of the genome, using lengths of alignments to the reference instead of contig lengths), and comparable or fewer misassemblies (Fig. 1; Supplementary Figs S4–S10;Supplementary Tables S3–S11). Notably, ntJoin improves assemblies with initial contiguity in the kilobase range to reach megabase scale (NGA50 increases from 26.9 kb to 2.3 Mbp and 19.8 kb to 50.3 Mbp, for *C.elegans* and *H.sapiens* short-read assemblies, respectively, Supplementary Figs S4 and S6), while reducing the misassemblies by over a third (33.5 and 61.5%, respectively). This highlights the potential of ntJoin in improving fragmented draft assemblies. Compared to Ragout, ntJoin achieved NGA50 values 1.1- to 2-fold higher for the short-read ABySS assemblies tested, although Ragout did scaffold a long-read Shasta assembly to a 1.2-fold higher NGA50 (Fig. 1; Supplementary Figs S4 and S6) (Jackman *et al.*, 2017; Shafin *et al.*, 2019). However, the Progressive Cactus alignment required for Ragout was very computationally expensive, running for over four days for all human runs and using over 115 GB of RAM, compared to the human ntJoin runs, which finished in under 13 min and used <11 GB of RAM. ntJoin was also faster than Ragoo in all tests, from 1.4 times faster for the Shasta assembly up to 35.8 times faster for the more fragmented *H.sapiens* ABySS assembly (Fig. 1; Supplementary Figs S4 and S6). ntJoin places 86.3–99.3% of the input assembly in scaffolds, a proportion that is very similar to both Ragout and Ragoo. Sequences may not be placed in scaffolds if they are too short as compared to the user-set window size or if the chosen reference is too divergent. While Ragoo uses a constant gap size between joined contigs (default 100 bp), ntJoin and Ragout estimate the gap sizes based on the reference, as evident from the total gap sizes in the Ragoo scaffolded assemblies being significantly smaller than both ntJoin and Ragout (Supplementary Tables S4–S15).

ntJoin can also improve draft assemblies when contiguous assemblies are available for the same species. This would be the typical use case in a project that uses multiple sequencing platforms for hybrid assembly. In Figure 1, a short-read ABySS assembly was scaffolded using a long-read Shasta (Shafin *et al.*, 2019) assembly. By retaining joins unique to the long and short-read sequences, ntJoin achieves an NGA50 higher than the baseline Shasta assembly. ntJoin provides an alternative assembly pipeline, where the structure of a long-read assembly informs the placement of short-read assembly sequences, precluding the need for polishing a long-read assembly with short reads. This approach can produce assemblies with high contiguity and base accuracy—particularly important for downstream genome annotation (Supplementary Tables S12–S15). On this data, neither Ragout nor Ragoo yield assemblies with a similarly high NGA50 length, and both require more time and memory compared with ntJoin.

The ntJoin approach also extends to scaffolding assemblies of different species, as demonstrated by scaffolding the saltwater and gharial crocodile assemblies using the American alligator genome as reference (Supplementary Tables S16 and S17). In our tests, the NG50 length of the crocodile assemblies increased to 14.44 and 12.92 Mbp for saltwater and gharial crocodiles (baseline NG50 = 0.14 and 0.07 Mbp), with a corresponding increase in BUSCO (Simão *et al.*, 2015) gene completeness of 2.6 and 9.5%, respectively. This demonstrates that ntJoin can still leverage synteny between these target and reference assemblies despite the species having diverged around 80 million years ago (Delsuc *et al.*, 2018).

There is some inherent reference bias in any reference-based assembly tool, and the user must consider this when designing their experiment, including in choosing the reference assembly. Here, we demonstrate the utility of ntJoin in fitting an input target assembly to the structure of the reference, which corrects misassemblies but also potentially breaks certain large structural variants. Similarly, the comparator tools were run in modes which can also cut the input contigs. While this mode will not break all structural variations (Supplementary Table S18), to avoid breaking/cutting the input contigs the ntJoin parameter ‘no_cut=True’ can be specified, which prevents erasing any existing structural variation in the target assembly (Supplementary Fig. S11).

In conclusion, ntJoin performs minimizer graph-based scaffolding quickly and with a small memory footprint, while still producing chromosome-level contiguity. As demonstrated, it is a flexible, alignment-free scaffolding tool that can be used in a number of different applications, including hybrid assembly and population genomics research.

## Funding

This work was supported by Genome BC and Genome Canada [243FOR, 281ANV]; and the National Institutes of Health [2R01HG007182-04A1]. The content of this article is solely the responsibility of the authors, and does not necessarily represent the official views of the National Institutes of Health or other funding organizations.

*Conflict of Interest*: none declared.

## Supplementary Material

btaa253_Supplementary_Data
